# Combined HIV Adolescent Prevention Study (CHAPS): comparison of HIV pre-exposure prophylaxis regimens for adolescents in sub-Saharan Africa—study protocol for a mixed-methods study including a randomised controlled trial

**DOI:** 10.1186/s13063-020-04760-x

**Published:** 2020-10-30

**Authors:** S. Nash, J. Dietrich, A. S. Ssemata, C. Herrera, K. O’Hagan, L. Else, F. Chiodi, C. Kelly, R. Shattock, M. Chirenje, L. Lebina, S. Khoo, L-G Bekker, H. A. Weiss, C. Gray, L. Stranix-Chibanda, P. Kaleebu, J. Seeley, N. Martinson, J. Fox, on behalf of the CHAPS team, Nadia Ahmed, Bernice Alinde, Alieu Amara, Millicent Atujuna, Linda-Gail Bekker, Francesca Chiodi, Mike Chirenje, Janan Dietrich, Jeffrey Dorfman, Laura Else, Julie Fox, Clive Gray, Christian Holm Hansen, Carolina Herrera, Stefanie Hornschuh, Ayoub Kakande, Pontiano Kaleebu, Charles Kelly, Saye Khoo, Mamkiri Khunwane, Limaktso Lebina, Joseph Makhura, Nomvuyo Mangxilana, Neil Martinson, Richard Muhumuza, Freddie Mukasa Kibengo, Gertrude Mutonyi, Stephen Nash, Teacler Nematadzira, Lumka Nobula, Kyle O’Hagan, Geoffrey Odoch, Natasha Pilay, Elzette Rousseau, Eugene Ruzagira, Ntombexolo Seatlholo, Janet Seeley, Thabiso Seiphetlo, Jennifer Serwanga, Robin Shattock, Andrew S. Ssemata, Lynda Stranix-Chibanda, Gugulethu Tshabalala, Helen Weiss

**Affiliations:** 1 London School of Hygiene and Tropical Medicine, London, UK; 2 University of the Witwatersrand Perinatal HIV Research Unit, Johannesburg, South Africa; 3 MRC/UVRI Uganda Research Unit On Aids, Entebbe, Uganda; 4 Imperial College London, London, UK; 5 University of Cape Town, Cape Town, South Africa; 6 University of Liverpool, Liverpool, UK; 7 Karolinska Institutet, Solna, Sweden; 8 King’s College London, London, UK; 9 University of Zimbabwe, Harare, Zimbabwe; 10 Desmond Tutu HIV Foundation, Cape Town, South Africa; 11 University of Zimbabwe College of Health Sciences, Harare, Zimbabwe; 12 London School of Hygiene, London, UK

**Keywords:** HIV, PrEP, Uganda, South Africa, Zimbabwe, Adolescents

## Abstract

**Background:**

HIV remains a major public health issue, especially in Eastern and Southern Africa. Pre-exposure prophylaxis is highly effective when adhered to, but its effectiveness is limited by cost, user acceptability and uptake. The cost of a non-inferiority phase III trial is likely to be prohibitive, and thus, it is essential to select the best possible drug, dose and schedule in advance. The aim of this study, the Combined HIV Adolescent PrEP and Prevention Study (CHAPS), is to investigate the drug, dose and schedule of pre-exposure prophylaxis (PrEP) required for the protection against HIV and the acceptability of PrEP amongst young people in sub-Saharan Africa, and hence to inform the choice of intervention for future phase III PrEP studies and to improve strategies for PrEP implementation.

**Methods:**

We propose a mixed-methods study amongst young people aged 13–24 years. The first component consists of qualitative research to identify the barriers and motivators towards the uptake of PrEP amongst young people in South Africa, Uganda and Zimbabwe. The second component is a randomised clinical trial (ClinicalTrials.gov NCT03986970, June 2019) using a novel ex vivo HIV challenge method to investigate the optimal PrEP treatment (FTC-TDF vs FTC-TAF), dose and schedule. We will recruit 144 amongst HIV-negative uncircumcised men aged 13–24 years from voluntary male medical circumcision clinics in two sites (South Africa and Uganda) and randomise them into one of nine arms. One group will receive no PrEP prior to surgery; the other arms will receive either FTC-TDF or FTC-TAF, over 1 or 2 days, and with the final dose given either 6 or 20 h prior to surgery. We will conduct an ex vivo HIV challenge on their resected foreskin tissue.

**Discussion:**

This study will provide both qualitative and quantitative results to help decide the optimum drug, dose and schedule for a future phase III trial of PrEP. The study will also provide crucial information on successful strategies for providing PrEP to young people in sub-Saharan Africa.

**Trial registration:**

ClinicalTrials.gov NCT03986970. Registered on 14 June 2019

**Supplementary information:**

**Supplementary information** accompanies this paper at 10.1186/s13063-020-04760-x.

## Background

HIV remains a major public health issue, especially in sub-Saharan Africa (SSA). Globally, there were an estimated 4700 new infections every day in 2018, with around 2000 of these in Eastern and Southern Africa [1]. A key goal towards ending the epidemic is the UNAIDS 90-90-90targets, set in 2014, that at least 73% of people living with HIV should achieve viral suppression by 2020 and 86% by 2030. Despite progress towards these goals, new strategies to protect against HIV infection are urgently needed.

Daily pre-exposure prophylaxis (PrEP) is highly effective when adhered to, but its effectiveness is limited by cost, user acceptability and uptake [2]. Event-based PrEP, defined as taking tablets before and after insertive sex, has been shown to be highly effective in men who have sex with men (MSM), but roll-out has been limited by lack of data in other populations and concerns about regime complexity and burden. The IPERGAY randomised controlled trial (RCT) demonstrated that PrEP, in the form of FTC-TDF (Truvada), reduced the rate of infection by 86% (95% CI 40–98%) amongst men who have unprotected receptive and insertive anal sex with men [3]. This trial used a schedule of two pills 2–24 h before sex, a third pill 24 h after the previous dose and a final pill 24 h later.

Tenofovir alafenamide (TAF) is a newer version of TDF with fewer renal and bone side effects than TDF and, in combination with FTC, has a smaller pill size than FTC-TDF. In vitro data show concentrations of TFV-diphosphate in PBMCs are 8- to 10-fold higher following oral FTC-TAF compared to oral FTC-TDF. However, the optimum regime for insertive sex is not known and could be at a lower dose than for receptive sex given the lower transmission risk. This provides a rationale for exploring whether lower doses of FTC-TAF are required for on-demand PrEP compared with daily PrEP [4]. Evidence for the efficacy of novel PrEP regimes would require a non-inferiority randomised controlled trial (RCT) with the standard FTC-TDF regimen as the comparison arm. However, due to the high efficacy of FTC-TDF, a very low incidence of HIV infection would be expected in the FTC-TDF arm, with consequent large sample sizes. The cost of such a phase III trial is prohibitive [5], and hence, it is unlikely that more than one candidate drug, dose and schedule will be tested in one trial. Therefore, it is essential to select the best possible candidate in advance of a phase III RCT.

An innovative solution is the use of ex vivo challenge, which in this context uses tissue biopsies taken from an individual who has received HIV prevention medication in vivo. The freshly resected tissue is then exposed to HIV in a laboratory setting. After a washout, the tissue supernatant is cultured for 3 weeks, with culture supernatants harvested to establish the level of HIV infection. The most commonly used endpoint for cervical, rectal and vaginal tissue has been levels of p24 antigen in culture supernatant [6].

Our proposed study, the Combined HIV Adolescent PrEP and Prevention Study (CHAPS), is a mixed-methods research programme which aims to inform the choice of intervention for future phase III PrEP studies in young people in sub-Saharan Africa and to improve strategies for PrEP implementation.

## Methods

The CHAPS study will consist of a social science study and an RCT. The social science components will use individual interviews, group discussions and quantitative surveys to explore the preferences of PrEP dosing, schedule, cost and access. The RCT will investigate the optimum on-demand PrEP dose and schedule for insertive sex of FTC-TDF and FTC-TAF using an ex vivo model of HIV infection in resected foreskins from men recruited for voluntary medical male circumcision (VMMC). This strategy ensures that the study will be relatively quick and cheap and will not expose men to any additional HIV risk. The study will start with the social science qualitative component, followed by the quantitative survey and RCT.

### Settings

The study will take place in four sites: Soweto and Cape Town in South Africa, Entebbe in Uganda and Chitungwiza in Zimbabwe. The RCT will take place only in Soweto and Entebbe.

In South Africa, the study will be conducted by the Perinatal HIV Research Unit at Wits University and the Desmond Tutu HIV Centre in Soweto and Cape Town. Soweto is located in the south-west of Johannesburg city, in Gauteng province, where the estimated HIV prevalence is 15% amongst 15–49-year-olds. In Cape Town, the study will take place in the Klipfontein/Mitchells plain district, located in the southern part of the Cape Town Metro, where HIV prevalence is 24%, the highest in the Western Cape.

In Uganda, the study will be conducted around Entebbe town, in Wakiso district, 40 km south of the capital, Kampala. Participants will be recruited from amongst people living in fishing communities, where HIV prevalence is estimated at 27% amongst 15–49-year-olds [7] and 20% in 15–24-year-olds [8]. Research in Uganda will be conducted by the Medical Research Council/Uganda Virus Research Institute and London School of Hygiene & Tropical Medicine Uganda Research Unit.

In Zimbabwe, the study will be conducted by the University of Zimbabwe College of Health Sciences Clinical Trials Unit in Chitungwiza, a city in Harare Province about 25 km south of Harare. HIV prevalence is estimated to be 7.3% amongst 20–24-year-olds in this province [9].

### Social science methodology

There are three sections to the social science research: group discussions (GDs), in-depth interviews (IDIs) and quantitative surveys (QS). The GDs and IDIs will be used to collect exploratory qualitative data to assess the perceptions (barriers and motivators) towards daily and on-demand PrEP amongst HIV-negative young people aged 13–24 years. The quantitative survey data will be collected from the same population and will be used to investigate demographics and socio-behavioural risk factors related to PrEP preferences amongst young people.

#### Sample size and recruitment

We will recruit equal numbers of male and female volunteers aged 13–24 years and willing to take a HIV test. All participants will be recruited through purposive community sampling. Exclusion criteria will be positive HIV status (except for the survey in Uganda) and evidence of a psychological/psychiatric disorder that would invalidate informed consent. Parental waiver of consent was approved by ethical boards for use in Cape Town, Entebbe and Chitungwiza. Once recruited for one component of the research (GD, IDI, QS), an individual will not be eligible for any other component. For South Africa and Zimbabwe, participants will be recruited from community groups, schools, churches, bars, taxi ranks and other public meeting places. In Uganda, participants will be recruited at fish landing sites with information on the study being provided through local leaders, Village Health Teams and project mobilisers.

We will enrol up to 192 people for GDs (8 groups per country, with 6–8 participants per group) and 60 for IDIs (20 per country). In addition, we will conduct 20 IDIs amongst RCT participants (10 per country) and researchers. For the QS, we will enrol 1300 participants with an equal gender ratio and stratification for specific age groups, with more participants in Uganda (*n* = 500) than in the other countries (*n* = 400) due to the inclusion of people living with HIV. As the study is descriptive, no formal sample size calculation has been performed. Recruitment in SA will be split equally between the two sites.

#### Acceptability of PrEP data collection methods

Group discussions will be used to identify attributes relating to PrEP which will include frequency of administration (i.e. daily vs on-demand PrEP), dispensing site (i.e. ARV clinic, pharmacy), time to be spent obtaining PrEP, frequency of collecting PrEP and frequency of HIV testing associated with PrEP. Interview guides for GDs and IDIs will be used to investigate topics including attitudes towards daily and on-demand PrEP and any relation with HIV risk. Barriers (e.g. stigma related to taking ARV medication) and motivators (e.g. using PrEP for HIV prevention) to PrEP will be explored, and approaches will be developed to maximise adherence to avoid PrEP failure (and consequent drug-resistant HIV) and to retain people in PrEP programmes. The guides will address multiple delivery options for PrEP uptake (Table 1). The interview guide will be pilot-tested amongst adolescent Community Advisory Boards (CABs) in each country.

**Table 1:** Topics of inquiry for group discussions and in-depth interviews

Topic	Prompts
**PrEP characteristics**	PrEP attributes including side effects, effectiveness, dosage, cost, dispensing (where and who)
**Behavioural change after PrEP**	Own risk perceptions on HIV, peer sexual norms, knowledge about HIV and HIV risk, multiple partners and transitional sex, condom use, sexual disinhibition on PrEP use
**PrEP in general**	Knowledge, perceptions and preferences about PrEP
**Acceptability of PrEP**	Willingness to use and receptiveness towards PrEP, reasons to use or not use PrEP
**Facilitators and barriers**	Social and community concerns regarding PrEP
**Gender dynamics**	Disclosure to partners, perceptions of partners taking PrEP

The survey will consist of closed questions, and in Soweto and Cape Town, a discrete choice experiment (DCE) will also be included. These will be administered to participants using electronic tablets with the Open Data Kit (ODK) application. The survey will cover PrEP preferences, sexual behaviour, views on HIV, mental health and alcohol and drug use. The DCE will investigate participants’ preferences on daily or on-demand PrEP, cost and access to PrEP.

#### Analysis of social science data

GDs and IDIs will be transcribed and analysed using a framework analysis approach. This provides a highly systematic method of categorising and organising data according to key themes, concepts and emergent categories in grids or matrices. The aim is to link qualitative findings with quantitative research [10] to contrast and enhance findings collected quantitatively and to increase the validity of findings through triangulation [11].

Data analysis will be undertaken by experienced qualitative researchers who have been provided with specific training in framework analysis, to ensure a consistent approach to analysis. The first five transcripts from each site will be coded by at two researchers at each research site and assessed for consistency. Differences will be resolved and a list of final codes shared with all sites. The remaining transcripts will be coded by at least one research analyst at each study site.

To gain qualitative feedback on the clinical trial implementation and use/perceptions of PrEP, in-depth interviews conducted with participants and health workers during trial exit visits will be analysed using the same data analysis methods discussed above.

Survey responses will be tabulated by age group, sex and site. The initial results will show the proportions of responses in each category for the survey questions. The main contrasts we will investigate are between people who prefer on-demand PrEP with those who prefer daily PrEP, and the acceptability of oral PrEP. For these analyses, respondents who answer “not sure” or “no preference” will be excluded. We will conduct multivariable logistic regression to investigate which factors influence PrEP preference. All regression models will be adjusted for sex, age group and site.

#### Methodology for the randomised controlled trial

The intervention, conducted in Soweto and Entebbe, consists of either FTC-TDF or FTC-TAF, in various doses and schedules. The outcome will be HIV infection of the resected foreskin in an ex vivo challenge. The trial was registered with ClinicalTrials.gov on 14 June 2019, with identifier NCT03986970. Recruitment rates and safety will be overseen by an independent data safety monitoring committee (DSMC) who reports to the Trial Steering Committee (TSC) and comprises a statistician and two leading HIV prevention research clinicians. The DSMC will review the trial’s progress including recruitment, data quality, adherence to protocol treatment and follow-up, and safety outcomes. Members are independent from the sponsor and have no competing interests. They will meet once at the beginning of the trial, once after ten participants have completed the study and once approximately halfway through recruitment or after 6 months (whichever is the earliest). The committee may meet more frequently if it becomes necessary to do so. The TSC is the oversight body and meets at least annually. There are 5 voting independent members (including the Chair) and 4 voting non-independent members including the trial PIs. Based on the recommendations from the DSMC, possible decisions by the TSC include early stopping due, for example, to clear benefit or harm of a treatment, futility, insufficient recruitment or external evidence; stopping recruitment within a site; modifying target recruitment or pre-analysis follow-up, based on any developments; and sanctioning and/or proposing protocol changes.

The trial has independent audit monitoring which is independent from investigators and the sponsor. The trial is monitored externally throughout the study and occurs independently from investigators and the sponsor.

#### Recruitment

The target sample size is 144 men, with equal numbers in South Africa and Uganda. As the trial is descriptive, no formal sample size calculation has been performed. The sample size of 8 participants per country was based on previous ex vivo challenge models using rectal and vaginal tissue. As there was considerable tech transfer, we chose to carry out 8 per country instead of 8 total per group. Participants will be drawn from men attending one of two VMMC clinics (Chris Hani Baragwanath Academic Hospital, Soweto, South Africa, and Entebbe General Hospital, Entebbe) or volunteers from the surrounding communities. The eligibility criteria are age 13–24 years, HIV-negative, clinically eligible for circumcision, weigh more than 35 kg, have a haemoglobin more than 9 g/L and be able to understand and sign a written informed consent form (for participants under 18, the legal guardian must also sign). Participants must satisfy all eligibility criteria within 21 days prior to their circumcision visit. No personal identifiable information will be shared or used in the analysis.

#### Design and intervention

**Fig. 1:**
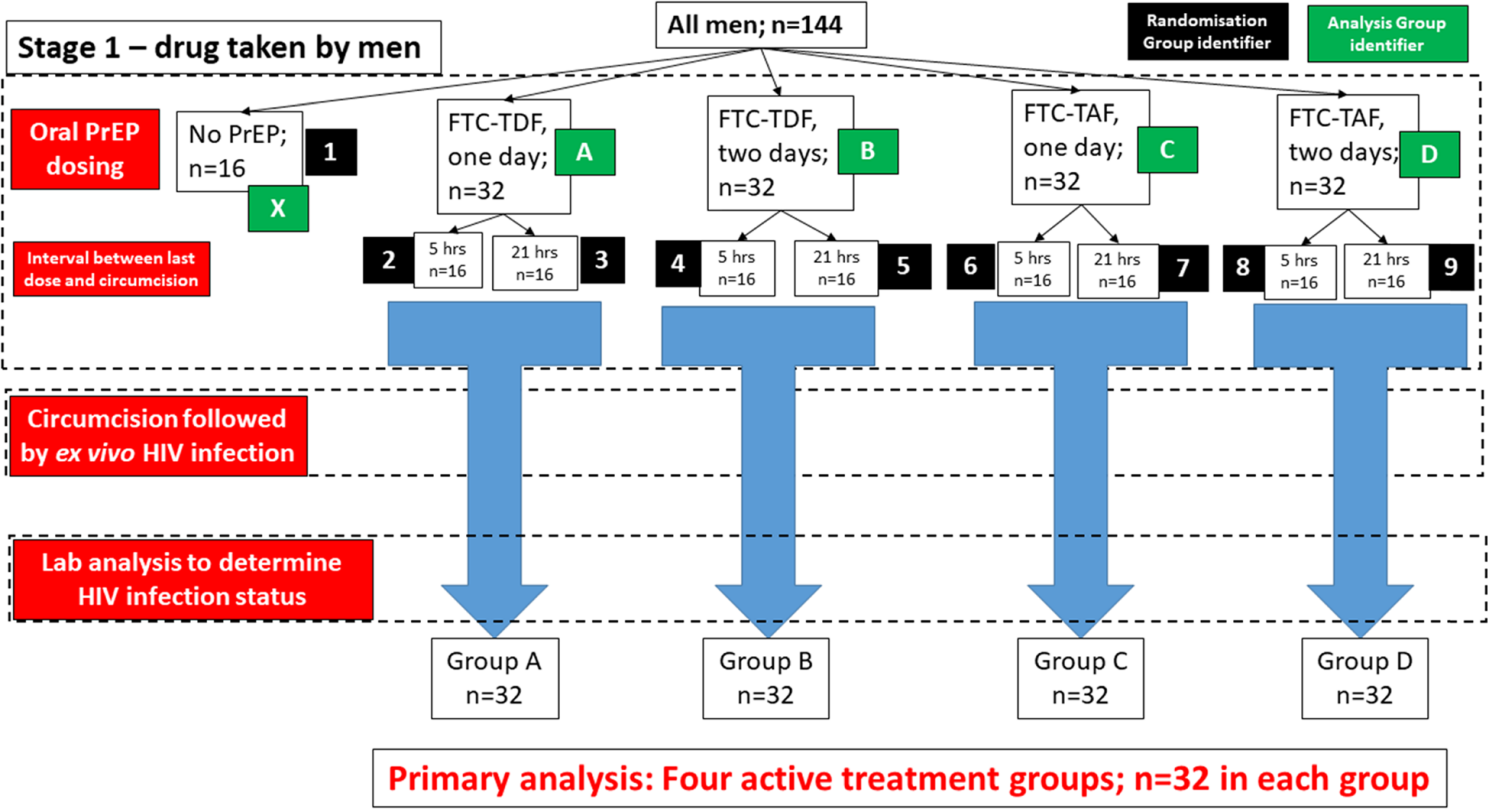
Flow diagram of participant and foreskin journey for primary analysis (oral PrEP only). The numbers in the black boxes indicate the randomisation groups—there are nine, with each man allocated to exactly one group, with each group equally likely. This figure shows recruitment from both sites; recruitment will be split equally between SA (*n* = 72) and Uganda (*n* = 72). The numbers in the green boxes refer to the analysis groups. The main comparison will be between groups A–D. Group X, men who do not receive PrEP, will be used as a comparison and to ensure that the ex vivo challenge model is successful

Enrolled participants will be randomised prospectively into one of nine groups in equal ratios, stratified by country (*n* = 144; 8 participants per group per country). Randomisation envelopes will be prepared by staff who were otherwise not involved in the CHAPS study. The randomisation will ensure there will be exactly eight participants per group in each country using block allocation, with blocks of size 9 and 18, prepared using the Stata command ralloc. During data analysis, the trial statistician will only be given dummy identifiers for each trial arm, so that summary statistics can be produced. The true group identifiers will only be revealed when the analysis is complete. Of the nine groups, eight will receive active treatment and one will be a control group where the men do not receive any PrEP (Fig. 1, Table 1). The eight treatment arms form a 2 × 2 × 2 factorial design, on three factors:

i. Drug: FTC-TDF or FTC-TAF
ii. Dose: either three tablets over 2 days or two tablets on 1 day
iii. Timing of VMMC: 5 or 21 h following the final dose of PrEP

The two drugs to be compared are FTC-TDF (also known as Truvada), which is a combination of emtricitabine (200 mg) and tenofovir disoproxil fumarate (300 mg, which is equivalent to 245 mg of tenofovir disoproxil) and FTC-TAF (known as Descovy), which is emtricitabine (200 mg) and tenofovir alafenamide (25 mg). All regimes will be taken as directly observed therapy doses administered by research staff. The timing of the VMMC will be either 5 or 21 h after the final dose of PrEP. There will be a 1-h permissible allowance either side of this time, so that the two windows will be 4–6 h or 20–22 h following the final PrEP tablet. Participants will also provide blood and urine samples, will be screened for STIs and will be invited to provide optional rectal, urethral and coronal sulcus swabs. If a participant is diagnosed as HIV-positive at screening, then they are referred immediately to services for follow-up and care. Any sexually transmitted infections diagnosed will be treated by the trial team.

At the VMMC visit or the final study visit (post-VMMC), all participants will complete a self- or interviewer-administered short exit survey using the electronic database, ODK and/or iDatafax. Questions will focus on motivation and concerns for trial participation, sexual behaviour, PrEP acceptability (for participants who took PrEP) and clinical trial experience. The survey will take approximately 5 min to complete. The exit survey will be translated into the main local languages of each country (South Africa: Zulu, Sesotho; Uganda: Luganda). Retention is promoted by the short duration of the trial. The intervention is the PrEP dose which is administered at most twice with the final dose either 5 h or 21 h before circumcision.

#### Ex vivo challenge

Foreskin tissue will be challenged ex vivo with HIV-1_BaL_ within 1 h of resection. Further post-exposure dosing (using the same drug taken by the participant) will be given to the resected foreskin following HIV exposure to simulate the effect offered by PrEP drugs taken after sex. The presence and level of HIV infection will be assessed by p24 antigen ELISA on foreskin culture supernatants from days 3, 7, 11 and 15 days. All cultures will be initiated immediately after circumcision in local laboratories within 30 min travel time from the VMMC clinic.

Each foreskin will be cut into segments (explants) to allow evaluation of each dose in three biological replicates. Results from explant challenge with the same viral titre will be combined by taking the mean, giving a single measurement for each participant. The outcome measures will be as follows:

1. p24 at day 15
2. Slope of p24 curve, days 3–15
3. p24 area under the curve, days 3–15

We will test two HIV challenge titres; a high titre (10^4^ TCID_50_/ml) normally used in pre-clinical assays in the laboratory and a low titre (10^2^ TCID_50_/ml) trying to mimic in vivo levels. Results from these two challenge titres will be considered separately and will not be combined. Data from the two countries will be combined for analysis. There will be four active treatment analysis groups (A–D, Table 2) and one control group (no PrEP; X in Table 2). We will combine the randomisation arms where men take the same drug, at the same dose, differing only in the time between the final dose and circumcision, unless the result of any particular outcome is markedly different (Table 3). Hence, there will be 32 men in each analysis group, except for the control group (*N* = 16).

**Table 2:** Table of participant randomisation groups and laboratory analysis of foreskins

Stage 1: Oral PrEP taken by men						Foreskin tissue divided in the lab	Stage 2: In vitro dosing of tissue in the laboratory		
Randomised arm	*N*	Oral PrEP analysis group	Oral drug	Number of PrEP doses	Interval between last oral PrEP and VMMC (h)		In vitro analysis group	In vitro drug	Interval between ex vivo HIV exposure and in vitro dose (h)
1	16	X	No PrEP	–	–		X-0	No PrEP	–
							X-TDF	FTC-TDF	18
							X-TAF	FTC-TAF	18
2	16	A	FTC-TDF	1	5		A-5-0	No PrEP	–
							A-5-TDF	FTC-TDF	18
3	16		FTC-TDF	1	21		A-21-0	No PrEP	–
							A-21-TDF	FTC-TDF	18
4	16	B	FTC-TDF	2	5		B-5-0	No PrEP	–
							B-5-TDF	FTC-TDF	18
5	16		FTC-TDF	2	21		B-21-0	No PrEP	–
							B-21-TDF	FTC-TDF	18
6	16	C	FTC-TAF	1	5		C-5-0	No PrEP	–
							C-5-TAF	FTC-TAF	18
7	16		FTC-TAF	1	21		C-21-0	No PrEP	–
							C-21-TAF	FTC-TAF	18
8	16	D	FTC-TAF	2	5		D-5-0	No PrEP	–
							D-5-TAF	FTC-TAF	18
9	16		FTC-TAF	2	21		D-21-0	No PrEP	–
							D-21-TAF	FTC-TAF	18

The in vitro analysis groups comprise three components: a letter indicating the stage 1 group, a number (5 or 21) indicating the interval between the last oral PrEP dose and circumcision and an item indicating in vitro dosing (where 0 = no dose). The exceptions are the group of men who did not receive oral PrEP (group X): these groups do not include the middle component

**Table 3:** Summary of randomisation arms

Randomised arm	Drug	Number	Dose 1	Dose 2 (24 h later) (± 1 h)	Interval between the last PrEP dose and surgery (h) (± 1 h)	Analysis group (Fig. 1)
1	Control	16	–	–	–	X
2	FTC-TDF	16	2 tablets	–	5	A
3	FTC-TDF	16	2 tablets	–	21	A
4	FTC-TDF	16	2 tablets	1 tablet	5	B
5	FTC-TDF	16	2 tablets	1 tablet	21	B
6	FTC-TAF	16	2 tablets	–	5	C
7	FTC-TAF	16	2 tablets	–	21	C
8	FTC-TAF	16	2 tablets	1 tablet	5	D
9	FTC-TAF	16	2 tablets	1 tablet	21	D

#### Qualitative interviews

We will conduct in-depth entry and exit interviews amongst a subset of RCT participants, to gain qualitative feedback on the clinical trial implementation and use of PrEP. Ten participants per site will be randomly selected. All research will be conducted in a local language of each country (South Africa: Zulu, Sesotho; Uganda: Luganda; Zimbabwe: English, Shona). Parental/guardian consent will be obtained for adolescents younger than 18 years. We will also conduct interviews with the trial health care providers to seek their perspective on the nature of PrEP experiences.

### Data management for the RCT

Protocol and data management training will be conducted prior to study initiation by the protocol team. Data will be collected at the site and recorded in participant chart binders, which will be reviewed for accuracy and completeness prior to participants leaving the clinic. Source data transcribed onto case report forms (CRFs) will be reviewed for completeness and transcription errors prior to data being entered into the REDCAP database. The REDCap database and programmed data cleaning queries and activities will be mapped in collaboration with the study team and outlined in the study data quality management plan. The REDCap database will have inbuilt edit checks and skip logic that is interactively activated on data entry prompting query resolution. Additionally, data will be extracted and reviewed bi-monthly using additional queries programmatically generated using the SAS software. These data queries will be sent to the sites by means of a quality control (QC) report every 2 weeks. Study management reports will also be circulated to the sites and the protocol team every week.

To ensure data confidentiality, participant identifiers will not be recorded on study CRFs and consequently not in the study database. REDCap is housed on secured servers and administratively managed by the University of the Witwatersrand in Johannesburg South Africa. User access and database permissions are controlled through unique individual login credentials that required to be updated every 90 days. The database will have an inbuilt audit trail as well, and study data will be shared only with the trial statistician. Study data will be password protected and encrypted before transfer.

Adverse events will be collected systematically (i.e. asked specifically of participants at follow-up visits). Unexpected adverse events will be collected on AE and SAE forms. SAEs will be reported in trial publications, by trial arm.

### Primary endpoint

**Fig. 2:**
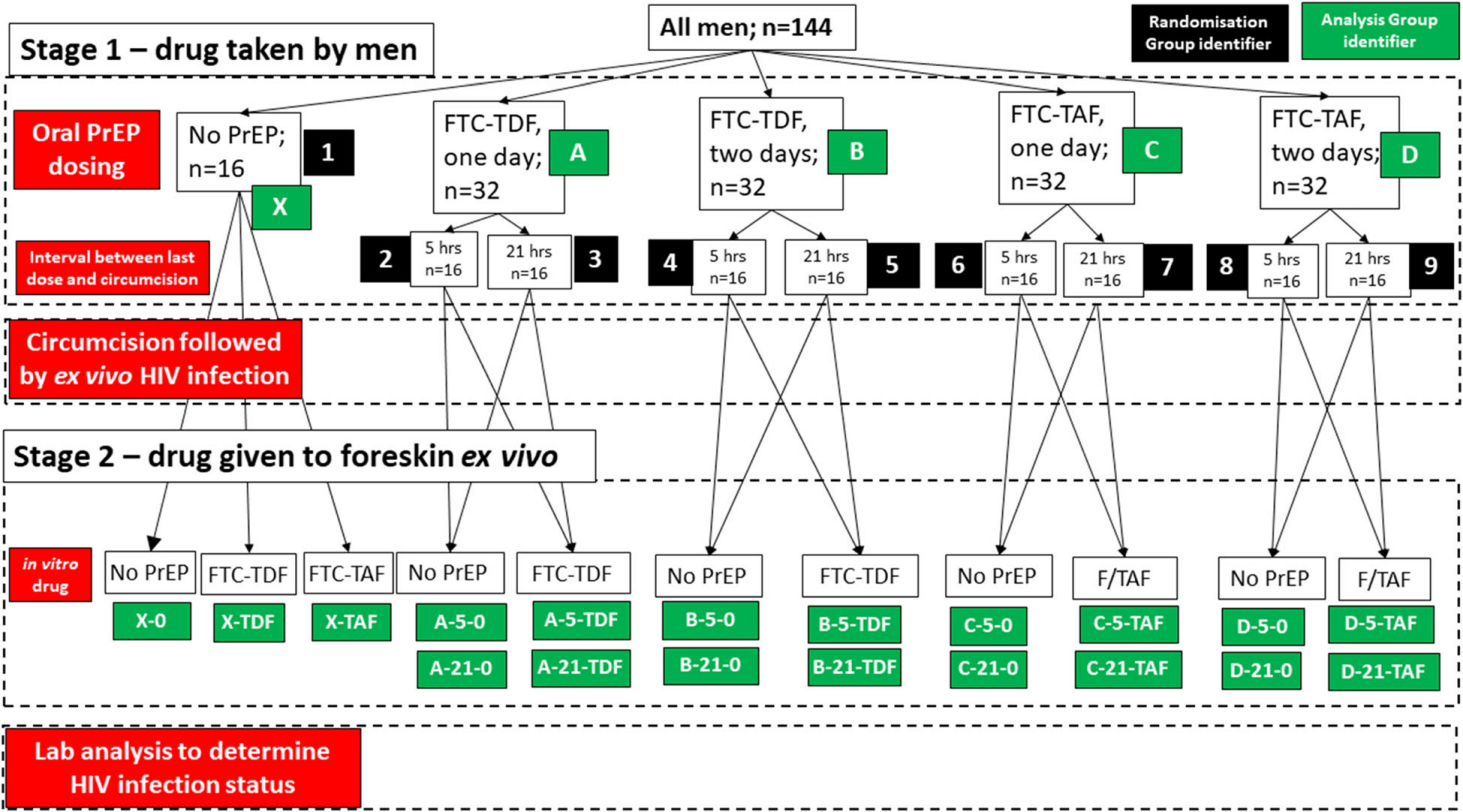
Flow diagram of participant and foreskin journey for oral PrEP, in vitro drug exposure and ex vivo HIV challenge. The analysis group identifiers in stage 2 comprise three components: a letter indicating the stage 1 group, a number (5 or 21) indicating the interval between the last oral PrEP dose and circumcision and an item indicating in vitro dosing (where 0 = no dose). The exceptions are the group of men who did not receive oral PrEP (Group X): these groups do not include the middle component

HIV infection will be assessed via measurement of p24 antigen. There will be three main HIV infection outcomes, as above, which will each be considered of equal weight when determining the efficacy of each treatment regimen. The primary comparison will be between groups A–D, as described in Figs. 1 and 2. These will be no formal statistical comparison between the groups as we are not powered to detect any particular difference, as the variability of the p24 responses is not known in this context. Instead, the means and standard deviations for each outcome in each group will be presented to allow comparison between the groups. We will present 95% confidence intervals to report the uncertainty of each estimate; however, we expect these intervals to overlap for all groups due to the small group sizes. The control group will be used to assess the effectiveness of the ex vivo challenge process.

Participants and care providers will not be blinded, but outcome assessors and data analysts will be. If the data safety monitoring board wish to see unblinded data, this will be provided by the independent statistician. The trial statistician will be given dummy group identifiers for each participant, so that means can be calculated for each group, but will not know which treatment each group received until group differences have been computed.

### Secondary endpoints

Additional analyses will be performed to assess the pharmacokinetic characteristics and investigate the added effect of post-exposure dosing of PrEP drugs. We will compare drug levels (TFV, FTC, TAF) in the plasma, peripheral blood mononuclear cells CD4 T cells (TFV-DP and FTC-TP) and rectal fluid. In addition, foreskin inflammation, microbiome phenotype and cellular activation of foreskin tissue following PrEP initiation will be investigated.

### Sample storage

Biological samples will be stored in a repository approved by the University of the Witwatersrand Bioethics Research Committee (Table 4). Any future use will be according to the requirements of the University of the Witwatersrand’s Human Research Ethics Committee (medical). In brief, the requirements are (1) all participants whose specimens are collected for repository storage must have provided consent for this, (2) repository samples may not only be used for a purpose that included in the original repository or trial/study informed consent, and (3) the University of the Witwatersrand HREC must be notified of the future use. There is no time-bound requirement placed on the investigators for repository storage.

**Table 4:** Sample collection and sample analysis

Specimen type	Volume/amount of sample	Analysis
**Safety blood**	4 ml EDTA	Haemoglobin
	5 ml SSAT	HIV
**Research blood**	51 ml ACD	Ex vivo challenge, drug levels, cytokines
**Rectal swab**	1 swab	Drug levels
**Urethral swab**	1 swab	Inflammation
**Coronal sulcus swab**	1 swab	Microbiome
**Foreskin**	Circumcised foreskin	Ex vivo challenge, drug levels, inflammation and microbiome
**Urine**	5 ml	Drug level
	5 ml	STI screen

### Trial status

**Fig. 3:**
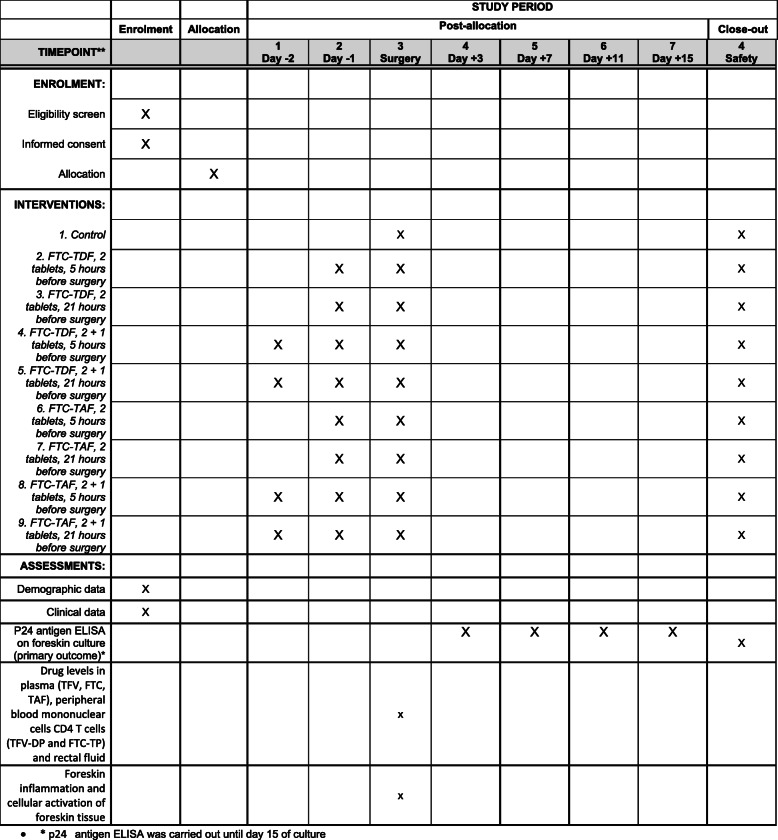
SPIRIT figure. *p24 antigen ELISA was carried out until day 15 of culture

Recruitment commenced in South Africa on 11 November 2019. Recruitment was hoped to start in Uganda in January 2020. We expect to complete the recruitment in both sites in August 2020. South Africa is operating under version 1.0 of the protocol, dated 8 August 2018. Uganda is using a slightly amended version, 1.2 (12 November 2018) which includes changes requested by the local ethics committee to account for site-specific procedures around hospitalisation and partner notification. A SPIRIT figure is included as Fig. 3, with a checklist included as a supplementary file.

### Dissemination plans

A whole or part of this trial results will be communicated, orally presented and/or published in appropriate scientific journals. Full anonymity of participant’s details will be maintained throughout. Participants wanting to see the results of the trial can request a copy of the article from the investigators once it has been published. We will follow standard authorship eligibility guidelines and will not use professional writers. The full protocol, participant-level dataset and statistical code will be publicly available on demand through the LSHTM Data Compass platform.

## Discussion

This innovative mixed-methods, multi-country study will assess the effectiveness and acceptability of PrEP medication amongst young people in sub-Saharan Africa. The use of ex vivo challenge will enable us to quickly and affordably provide a first assessment of the efficacy of a range of PrEP drugs, doses and schedules for insertive sex. The results from the RCT, combined with the rich qualitative data from the social science study, will inform the planning and design of a future phase III trial of PrEP regimes.

The in-depth interviews, group discussions and qualitative survey will provide detailed, contextual information on the ways in which people may take PrEP. By focusing on sexually active young people, we plan to design a future trial of PrEP which will be welcomed by this key population. The inclusion of a discrete choice experiment will allow us to assess which factors of the delivery of PrEP are most important to young people.

A critical feature of this study is the interplay between the social science and RCT components. In particular, the experience of recruitment for the survey will help with recruitment for the RCT, both by helping our teams on the ground learn by experience, and by building links with the host communities and sensitising local residents to the trial. One of the key strengths of the trial design is the short duration of participant contact. This reduces the chance of loss to follow-up and helps to provide a quick answer for the scientific question. In addition, the skill transfer of ex vivo challenge to the laboratories in South Africa and Uganda provides significant capacity building. Unlike studies with HIV incidence as an outcome, no participants will be exposed to additional risk of HIV due to group allocation, and we will make it clear to men that the PrEP doses given will not confer protection against HIV. The combination of RCT and in-depth qualitative research will allow for informed decision-making about the next steps of PrEP dosing and schedule.

In the survey, we accept that the proportions reported for each group are unlikely to be generalisable to the wider population because our sampling was purposeful, rather than random, and it is likely that we will have recruited young people with different attitudes towards PrEP and HIV. The lack of formal statistical testing is justified by the design and aims of the RCT. As the aim of the study is to provide guidance for the design of future studies, we will assess the results of the trial in the light of findings from the social science element, to assess which combination of drug, dose and timing will likely be most effective and acceptable to the target populations. In an era when efficacy studies for HIV protection are becoming prohibitively large and expensive, it is not feasible to study multiple regimens in a phase III setting. Evidence from phase 2 studies of this nature will maximise the chance of identifying the most efficacious dosing strategy that can progress into efficacy trials. This factorial design using a surrogate endpoint can be used to efficiently select drug combinations and dosing regimens for testing in phase III, while obviating the unnecessary costs of futile regimens. We see this study design as vital to providing rapid stop-go decisions for a range of treatment options in a cost-effective and timely way. The results from this study should mean that the risks to participant in a future trial are minimised by selecting the drug, dose and schedule which will offer the best protection. The knowledge we discover should be critical to the design of future trials.

## Supplementary Information

**Additional file 1:.** SPIRIT 2013 Checklist: Recommended items to address in a clinical trial protocol and related documents.

## Data Availability

Trial data will be available on request from LSHTM Data Compass.
